# A semi‐automated observation approach to quantify mouse skeletal muscle differentiation using immunohistochemistry

**DOI:** 10.14814/phy2.70330

**Published:** 2025-04-14

**Authors:** Kenta Shimizu, Yamato Yoshida, Kenshin Iwasa, Yasuyuki Fujii, Ursula M. Jacob, Tilman Fritsch, Ali S. Abdelhameed, Vittorio Calabrese, Naomi Osakabe

**Affiliations:** ^1^ Systems Engineering and Science, Graduate School of Engineering and Science Shibaura Institute of Technology Saitama Japan; ^2^ Department of Bioscience and Engineering, Faculty of System Science and Engineering Shibaura Institute of Technology Saitama Japan; ^3^ Healthcare AG Zurich Switzerland; ^4^ NAM Institute Salzburg Austria; ^5^ Department of Pharmaceutical Chemistry College of Pharmacy, King Saud University Riyadh Kingdom of Saudi Arabia; ^6^ Department of Biomedical and Biotechnological Sciences University of Catania Catania Italy

**Keywords:** BrdU, diffrentation, immunohistochemistry, Pax‐7, semi‐automated quantification, skeletal muscle satellite cells

## Abstract

Histological analysis is vital for understanding skeletal muscle diseases. However, quantifying data requires much effort, so automation is expected to reduce workload. The present study proposes a semi‐automated method to quantify expressed paired box protein (Pax‐7) /bromodeoxyuridine (BrdU)‐positive cells. Soleus muscle was harvested from mice 2 weeks after oral administration of the epicatechin tetramer cinnamantanin A2 (A2), known to induce skeletal muscle hypertrophy. Before the necropsy, mice were treated with BrdU to facilitate cell tracking. For histological examination, frozen sections were stained with hematoxylin and eosin (HE) to measure cell size by cross‐sectional area (CSA) and were immunostained with anti‐BrdU and anti‐Pax‐7 antibodies. Treatment with A2 caused a shift in the CSA distribution curve towards larger values, thus revealing an increase in muscle size. The analysis of BrdU/Pax‐7 positive cells, performed both manually and semi‐automatically, revealed a slight increase with A2 treatment, while Pax‐7 positive cells remained unchanged. Correlation between manual and semi‐automated analysis showed a coefficient of determination of 0.7132, indicating a significant reduction in analysis time by approximately 20 times. This study highlights the effectiveness of semi‐automated histological analysis in skeletal muscle research and provides a practical solution to increase the efficiency of muscle regeneration evaluation.

## Graphical abstract

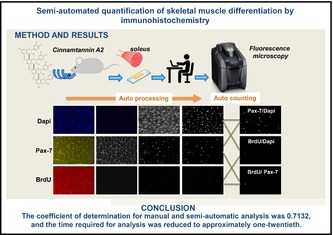

In this study, we developed a semi‐automated method to quantify Pax‐7 positive cells in skeletal muscle. The results of the developed semi‐automated method showed good correlation with the manual method, and the analysis time was reduced to one‐twentieth of the manual method.

## 1 INTRODUCTION

Histological analysis of skeletal muscle is essential to understanding the effects of exercise, nutrients, and phytochemicals on skeletal muscle and pathological conditions. Quantification of experimental datasets typically requires intensive human effort over weeks or months. Consequently, many automated platforms have been developed to unbiasedly assess skeletal muscle cross‐sectional area by muscle fiber type (Babcock et al., 2020; Desgeorges et al., 2019; Mayeuf‐Louchart et al., 2018; Smith & Barton, 2014; Waisman et al., 2021), central core (Babcock et al., 2020; Smith & Barton, 2014), and central core fibers (Smith & Barton, 2014).

Skeletal muscle also has an excellent regenerative capacity due to the activity of skeletal muscle resident stem cells called muscle satellite cells (Fukada et al., 2020). The plasticity of skeletal muscle adapts muscle fiber size in response to external stimuli, endogenous factors, or physical activity. Previous studies in experimental animal models have confirmed that skeletal satellite cells normally exist in a quiescent state and can become activated, proliferate, and differentiate when stimulated. Satellite differentiated myoblasts can either fuse to form new muscle fibers or bind to existing muscle fibers and donate their nuclei to the fibers, thereby increasing skeletal muscle mass. They can also return to a quiescent state and self‐renew to replenish the satellite cell pool. (Ismaeel et al., 2024; Snijders et al., 2015). In addition, human studies conducted over the past decade have also demonstrated similar roles for muscle satellite cells (Snijders et al., 2015). The paired box transcription factor (Pax)‐ 7 is expressed in a characteristic manner of muscle satellite cells. It drives the myogenic program by regulating the expression of myogenic regulatory factors, myogenic differentiation (MyoD), and myogenic factor (Myf) 5, whose expression is enhanced and differentiates into myotubes by promoting the expression of myogenin and myogenic regulatory factors (MRF) 4, which are essential factors for myogenic differentiation (Jiang et al., 2024; Seale et al., 2000). In evaluating skeletal muscle plasticity, assessing the expression of myogenic regulatory factors through fluorescent immunostaining is necessary.

The ingestion of polyphenols such as quercetin (Chen et al., 2021) and theaflavin (Suzuki et al., 2021), as well as the tetramer catechin cinntamtannin A2 (A2) (Muta et al., 2023), has been shown to induce skeletal muscle hypertrophy similar to that observed with resistance and load‐bearing exercise (Abreu & Kowaltowski, 2020). These effects may be related to the differentiation and proliferation of satellite cells. Therefore, the development of automated quantification methods for Pax‐7, as one of the myogenic regulatory factors, has constituted a recent research focus (Lundquist et al., 2023; Rahmati & Rashno, 2021).

Here a semi‐automated quantitative method was developed to detect newly generated Pax‐7 positive cells in response to various interventions. This study aimed to observe musculoskeletal disorders caused by aging and the effectiveness of exercise interventions in a rational and early manner. The pretreatment of mice with bromodeoxyuridine (BrdU) in conjunction with immunostaining to detect Pax‐7 and/or BrdU enables a more comprehensive investigation of satellite cell proliferation and differentiation. The images obtained were uniformly corrected by computer to facilitate the detection of positive cells and were subsequently quantified automatically. The validity of the developed method was evaluated by observing the changes in cell neogenesis and satellite cells in the soleus muscle when mice were repeatedly administered A2.

## 2 MATERIALS AND METHODS

### 2.1 Materials

Cinntamtannnin A2(A2) was obtained from Phytolab GmbH & Co., KG (49870–79, Vestenbergsgreuth, Germany). BrdU was purchased from Abcam Limited (ab142567, Cambridge, UK).

### 2.2 Animal study

The study was approved by the Animal Care and Use Committee of the Shibaura Institute of Technology (Permit Number: AEA23008, 2023). All mice received humane care under the National Institutes of Health guide for the care and use of laboratory animals in this institution. All surgery was performed under anesthesia, and all efforts were made to minimize suffering. Male C57BL/6J mice aged 8 weeks were obtained from Charles River Laboratories Japan, Inc. (C57BL/6JJcl, Tokyo, Japan). The mice were kept in a room with controlled lighting (12/12 h light/dark cycles) at a regulated temperature of 23–25°C. A certified rodent diet (MF®, for breeding) was obtained from Oriental Yeast Co., Ltd., Tokyo, Japan. After 2 weeks of acclimatization, the mice were randomly divided into two groups: a control group (distilled water, *n* = 9) and a 25 μg/kg A2 administration group (n = 9), and each group was administered by gavage for 2 weeks. 50 mg/kg BrdU was administered to all experimental animals intraperitoneally for 3 days before dissection after decapitation. The soleus muscles on both sides of the body were harvested and weighed. Given the presence of a heterogeneous mixture of muscle fiber types in mouse soleus, a characteristic that renders it an optimal model for the development of a quantitative methodology, four sections were obtained from the left soleus muscle of each animal. Three of these were subjected to immunostaining, while one was examined for hematoxylin and eosin (HE) staining. The right soleus muscle was preserved as a reserve.

### 2.3 Immunohistochemistry

For immunohistochemistry, the soleus was blocked with FSC 22 Blue (3,801,481; Leica Biosystems, Nussloch, Germany), frozen with isopentane (168–09195, Fuji Film Wako Chemical Corporation, Tokyo, Japan) on dry ice, and stored at −80°C. To prepare frozen sections, we cut 8‐μm‐thick slices of the soleus with a cryostat (CM1950; Leica Biosystems). To perform immunohistochemistry, we fixed sections with ice‐cold methanol for 10 min. Next, the sections were washed twice with Tris‐buffered saline combined with 0.1% Tween 20 for 5 min. Then, sections were incubated with blocking reagent (06349–64, Blocking One Histo; Nacalai Tesque, Inc. Kyoto, Japan) for 10 min at 4°C. The sections were incubated overnight at 4°C with the anti‐BrdU antibody (1:100, ab221240, Abcam), anti‐Pax‐7 antibody (1:100, ab221240, Abcam) and anti‐laminin antibody (1:200, L9393‐100UL, Sigma‐Aldrich, MO).

Next, sections were incubated at 4°C for 1 h with goat anti‐rabbit IgG H&L (1:200, Ab150077, Alexa Fluor®488; Abcam) or goat anti‐mouse IgG H&L (1:500, ab150120, Alexa Fluor®594). Then, the sections were mounted with VECTASHIELD Mounting Medium and counterstained with Dapi (H‐1200; Vector Laboratories, CA).

### 2.4 Hematoxylin eosin (HE) staining

Subsequently, serial sections were stained with hematoxylin and eosin (HE). Sections were immersed in 10% neutral buffered formalin (068–01663, Fujifilm Wako Pure Chemical Corporation, Tokyo, Japan) for 2 min, then washed and immersed in hematoxylin (121–09665, Fujifilm Wako Pure Chemical Corporation, Tokyo, Japan) for 90 sec. Sections were washed and immersed in 1% eosin (051–06615, Fujifilm Wako Pure Chemical Corporation, Tokyo, Japan) for 15 s, rinsed, dehydrated, immersed in xylene (241–0091, Fujifilm Wako Pure Chemical Corporation, Tokyo, Japan) for 3 min, and sealed with a soft mount (192–16,301, Fujifilm Wako Pure Chemical Corporation, Tokyo, Japan).

### 2.5 Microscopy

Sections stained by immunohistochemically were observed with a digital fluorescence microscope using a Z stack setting (BZ‐X800; Keyence Corporation, Osaka, Japan). Image acquisition was performed with full focus using a magnification of 40× and a resolution of 640 × 480 pixels. The analysis application BZ‐H4A (Keyence Corporation) was utilized. Dapi was detected using the BZ‐X filter Dapi, Pax‐7 was detected using the Leerer Filter für BZ‐X, and BrdU was detected using the BZ‐X filter TexasRed. The observation of the sections stained by HE was conducted utilizing a system biological microscope (CX41L, OLYMPUS CORPORATION).

### 2.6 Image processing

Fiji (https://imagej.net/software/fiji/, accessed on August 03, 2024), a macro function of the image analysis application ImageJ software, was used to quantify Pax‐7 and BrdU stained images saved as a tiff file. The code used for this process was shown in the supplemental code—Data S1.

**FIGURE 1:**
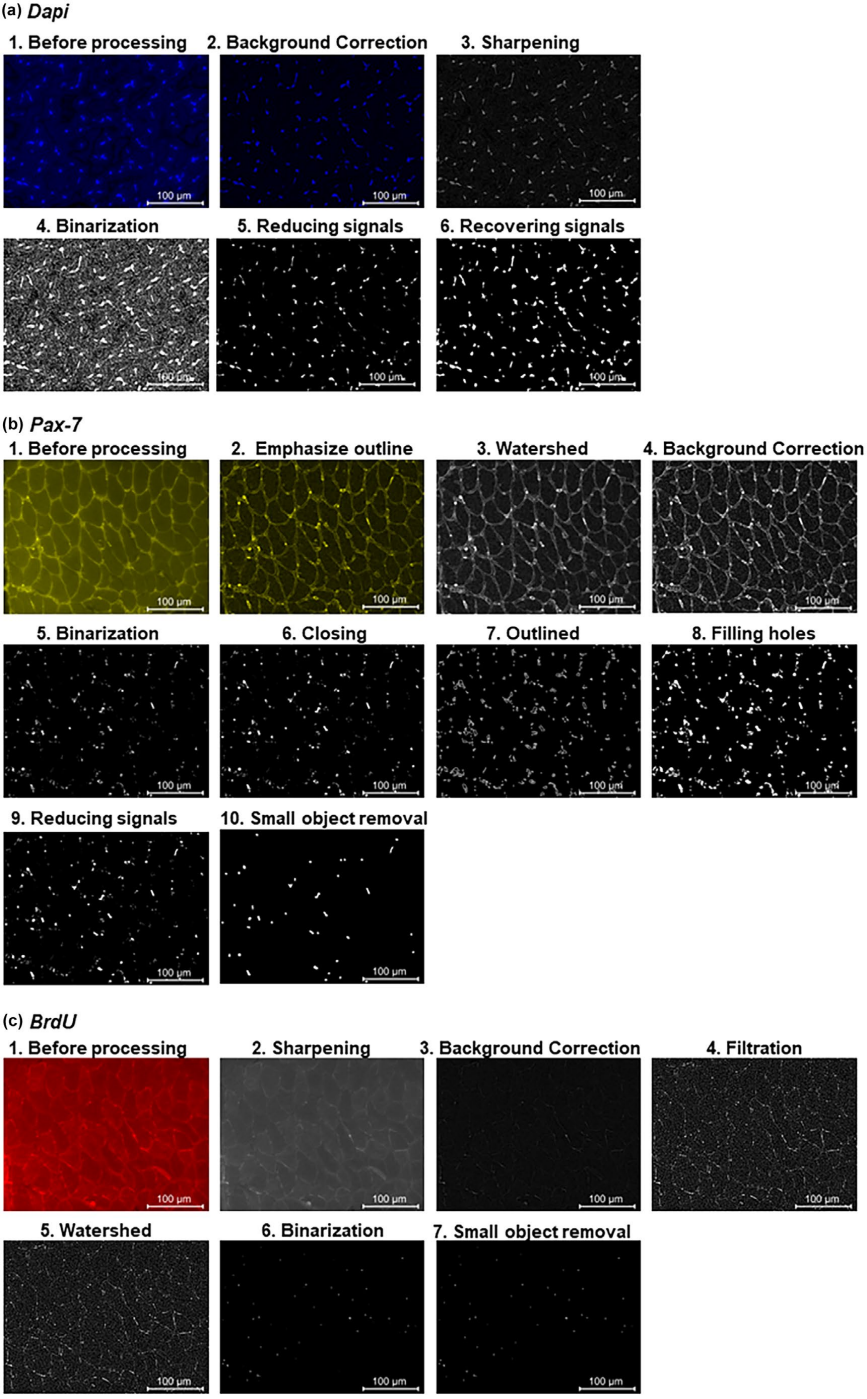
Image processing for Dapi (a), Pax‐7 (b), and BrdU (c) staining. (a) Dapi staining; 1. Before processing, 2. correction background (rolling ball method), 3. sharpening, 4. binarization (mean threshold method), 5. reducing signals, 6. recovering signals.(b) Pax‐7 staining; 1. Before processing, 2. emphasize signal outline correction, 3. watershed segmentation, 4. correcting background (rolling ball method), 5. Binarization (RenyEntropy method), 6. closing, 7. outlined, 8. filling holes, 9. reducing signals, 10. small object removal. (c) BrdU staining; 1. Before processing, 2. sharpening, 3. correcting background (rolling ball method), 4. filtration, 5. watershed segmentation, 6. binarization (Internode's method), 7. small object removal.

The obtained Dapi‐stained microscopic image is shown in Figure 1a1. In Dapi‐stained sections, the local fluorescence intensity inside myofibers can vary throughout the sample due to nonspecific staining and changes in tissue thickness. To suppress this variation, we adopted the rolling ball method to correct these backgrounds (Sternberg, 1983) (Figure 1a2), and set it to grayscale with the option in 8 bit. A sharpness filter was used to focus the signal (Figure 1a3) (Moles Lopez et al., 2013). Binarization was conducted with the mean threshold method, which calculates the image's average brightness value and is used as a threshold to convert objects in the image to white and the background to black (or vice versa) (Figure 1a4). After binarization, the extra pixels remaining around the signal were removed by reducing the object size (Figure 1a5). By expanding the signal's boundary and increasing the object size, the remaining object was the same size as before the shrinkage (Figure 1a6). The remaining signals were quantified as nuclei.

A typical Pax‐7 staining image is shown in Figure 1b1. Fiji's edge detection tool was used to emphasize the signal outline, and the image was then converted to 8‐bit (Figure 1b2). The luminance (Py) of each pixel was calculated as follows from the original luminance (Px) of each pixel and the minimum (a) and maximum (b) luminance in the image, and these results were used for watershed segmentation (Figure 1b3) (Vernon, 1991)

$$P_{y}=\left(P_{x}-a\right)\left(\frac{d-c}{b-a}\right)$$

Background correction was then performed using the rolling ball method (Figure 1b4). The obtained image was binarized using the method of RenyiEntropy, which is defined below. The probability distribution of the object (Babcock et al., 2020) and background (Waisman et al., 2021) in the image was calculated and used as a threshold (Figure 1b5) (Prewitt & Mendelsohn, 1966).

$$H_{T}^{\alpha }=\frac{1}{1-\alpha }\ln\sum_{\kappa =0}^{255}{\left(\mathrm{P\kappa}\right)}^{\alpha },\alpha \neq 1,\alpha >0$$

(1) $$H_{A}^{\alpha }\left(t\right)=\frac{1}{1-\alpha }\ln\sum_{i=0}^{t}{\left(\frac{P_{i}}{P\left(A\right)}\right)}^{\alpha }$$

(2) $$H_{B}^{\alpha }\left(t\right)=\frac{1}{1-\alpha }\ln\sum_{i=i+1}^{255}{\left(\frac{P_{i}}{P\left(B\right)}\right)}^{\alpha }$$

If a cavity was found inside the signal through these processes, after closing the opening of the signal (Figure 1b6), it was outlined (Figure 1b7) and the pixels in the cavity were filled in (Figure 1b8). By reducing the object size (Figure 1b9), the extra pixels remaining around the signal were removed (Figure 1b10). Finally, a small object was removed, and those larger than a certain size were quantified as Pax‐7.

A typical BrdU staining image is shown in Figure 1c1. The image was set to 8 bits, and then we used a sharpness filter to focus the signals (Figure 1c2). Next, we adopted the rolling ball method to correct the backgrounds (Figure 1c3) (Sternberg, 1983). Filtration was carried out using the Kernel's convolution processing method (Figure 1c4), and watershed segmentation was conducted as well as the Pax‐7 staining image as described above (Figure 1c5). The obtained image was binarized using the method of Intermodes (Figure 1c6) (Prewitt & Mendelsohn, 1966). Finally, isolated pixels were removed from the object (Figure 1c7), and those larger than a certain size were quantified as BrdU.

### 2.7 Quantification

The Fuji code used for quantification was shown in the supplemental code—Data S1.

**FIGURE 2:**
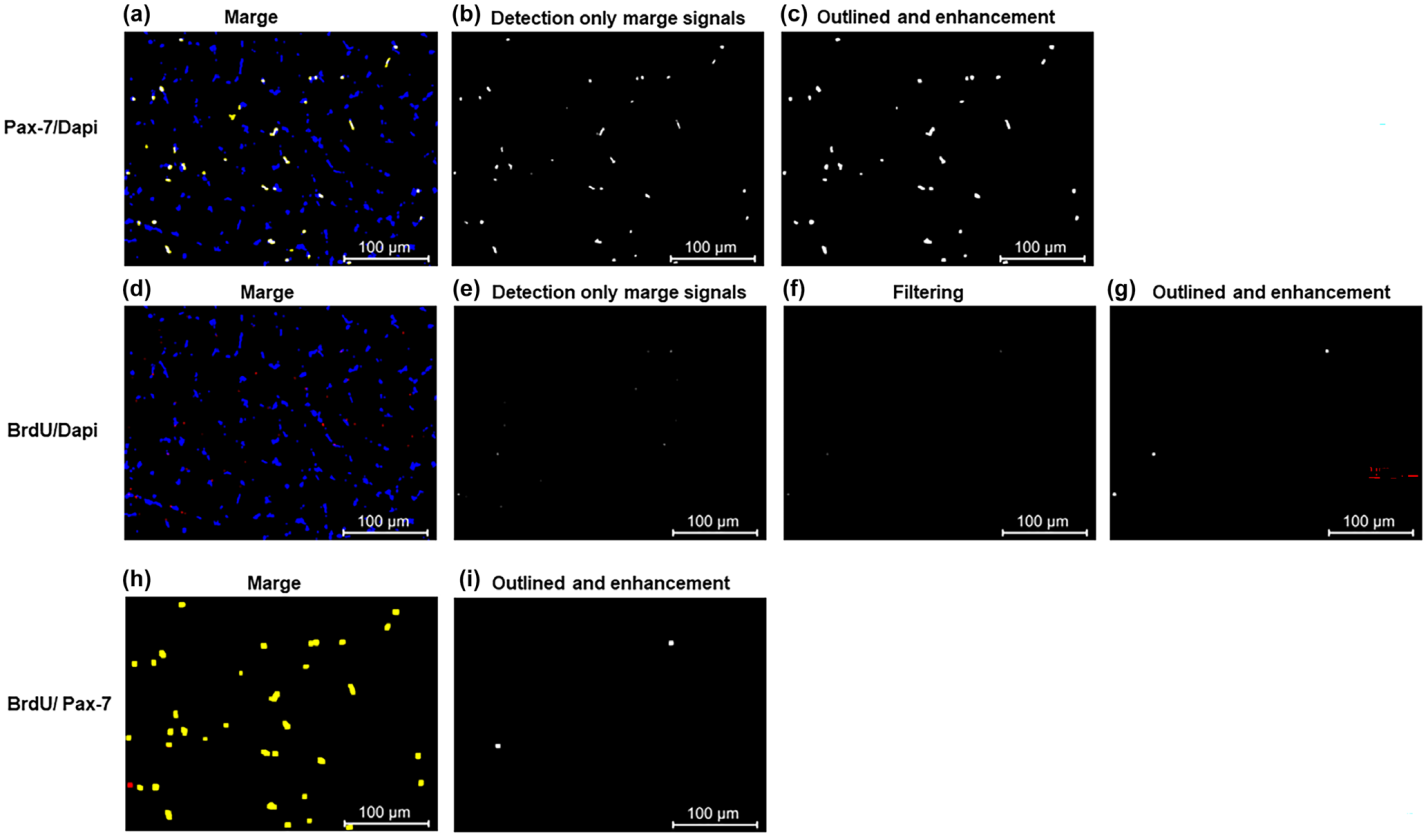
Image processing for quantification of Pax‐7/Dapi (upper panels), BrdU/Dapi (middle panels), or BrdU/Pax‐7 co‐positive cells (lower panels). Marge, (a); detection only marge signals, (b); outlined and enhancement of signals, (c) in Pax‐7/Dapi staining. Meerge, (d); detection only marge signals, (e); filtering, (f); outlined and enhancement of signals, (g) in BrdU/Dapi staining. Marge, (h); outlined and enhanced signals, (i) in BrdU/Pax‐7 staining.

The Dapi or Pax‐7 signals purified using the abovementioned method were merged (Figure 2a). The locations where both pixels were present were detected, and a new image was generated (Figure 2b). Subsequently, the border with the background was expanded (Figure 2c). The object size was inflated to prevent multiple expressions from being detected in the same nucleus. The number of signals in the generated image was counted as Pax‐7 expression cells.

The Dapi or BrdU signals purified using the method mentioned above were merged (Figure 2d). The locations where both pixels were present were detected, and a new image was generated (Figure 2e). Following this, a median filter was applied to replace the signal brightness with the median value of its surrounding area (Figure 2f). This process can prevent any incorrect detection of the background. Subsequently, the border with the background was expanded to prevent multiple expressions from being detected in the same nucleus (Figure 2g). The number of signals in the generated image was counted as BrdU expression cells.

The BrdU or Pax‐7 signals purified using the method mentioned above were merged (Figure 2h). The border with the background was expanded to prevent multiple expressions from being detected in the same nucleus (Figure 2i). The number of signals in the generated image was counted as co‐expression cells of BrdU/Pax‐7.

### 2.8 Statistical analysis and validation

All data were reported as mean ± standard deviation with individual data. Statistical analyses were performed by non‐parametric Mann–Whitney tests using the statistical software GraphPad Prism 10 (https://www.graphpad.com/features). The probability of *p* < 0.05 was considered significant.

## 3 RESULTS

**FIGURE 3:**
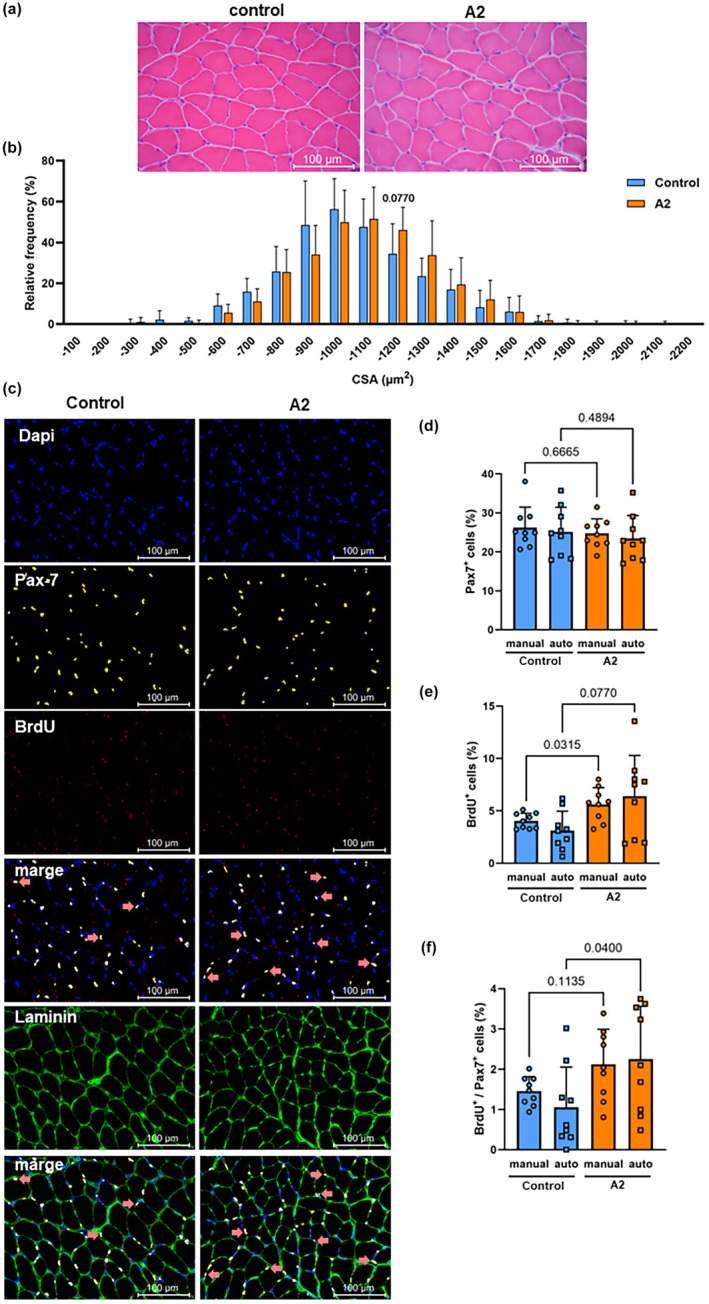
The results of cross‐sectional area (CSA) and immunohistochemical analysis in mouse soleus after control (distilled water) or administration of 25 μg/kg cinnamtannin A2 (A2) for 2 weeks. The image of HE staining, (a); results of CSA, (b); Image of Dapi, Pax‐7, BrdU, and marged of Dapi, Pax‐7, and BrdU, laminin, and marged of all was showed from top to bottom, (c); ratio of Pax‐7 positive cells analyzed by semi‐auto and manual, (d); ratio of BrdU positive cells analyzed by semi‐auto and manual, (e); ratio of BrdU/Pax‐7 co‐ positive cells analyzed by semi‐auto and manual, (f). Each value represents the mean and standard deviation (*n* = 9, each). Statistical analyses were performed by non‐parametric Wilcoxon and Mann–Whitney *U* tests.

The body and total soleus weight were 25.7 ± 1.6 (g) and 303.2 ± 27.4 (mg) for the control group, and 25.7 ± 1.4 (g) and 320.3 ± 17.2 for 4 (mg) the A2 group. There was no significant difference between the experimental groups. The HE‐stained image of the soleus and the results of the CSA measurement were shown in Figure 3a,b. As demonstrated in Figure 3a, the observation of the HE‐stained image suggested that the individual cells in group A2 exhibited a larger size compared to those in the control group. Furthermore, the measurement results indicated an upward shift in the peak muscle CSA in the A2 group compared to the control group, suggesting A2 increased muscle size (Figure 3b). The histochemical image of DAPI, Pax‐7, BrdU, and merged DAPI, Pax‐7, and BrdU, laminin, and merged of all was shown from top to bottom in Figure 3c. Figure 3d,e represented a comparison of the quantitative results of muscle differentiation markers in the soleus muscle after FL administration using the developed automated quantification method and the manual method. When comparing the results of the developed automated and manual quantification methods, there was no difference in the same group (*p* > 0.999). The number of Pax‐7 positive expressing cells did not change between the control group and the A2 group using either method (Figure 3d). The expression of BrdU tended to increase with A2 administration (Figure 3e). The number of BrdU and Pax‐7 co‐positive cells also increased or tended to increase with A2 administration (Figure 3f). Furthermore, fusion with laminin staining images showed that these BrdU and Pax‐7 co‐positive cells were in the niche between the basement membrane and sarcoplasmic membrane of muscle fibers.

**FIGURE 4:**
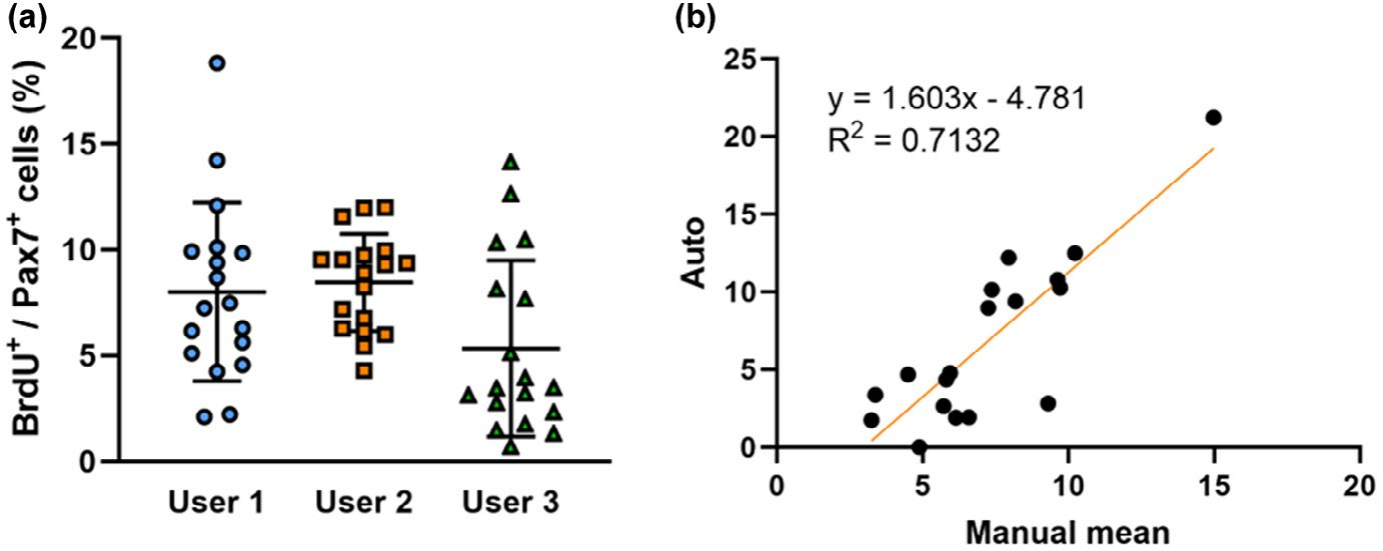
Comparison between manual and semi‐auto quantification on the ratio of BrdU/ Pax‐7 co‐positive cell in mouse soleus after control (distilled water) and administration of 25 μg/kg cinnamtannin A2 (A2) for 2 weeks. Distribution of each user's manual analysis results for all soleus (a, *n* = 18); correlation analysis results between semi‐automated and manual methods, (b, *n* = 18).

Figure 4 showed the validation of the developed automated method. Figure 4a shows the results of manual counting by three observers. Figure 4b showed the correlation analysis results between semi‐automated and manual processes. The regression equation based on the correlation analysis between the semi‐automated and manual methods was shown in Figure 4b. The coefficient of determination (*R* ^2^) was 0.7132. For the present sample size, it took about 1 month to quantify BrdU and Pax‐7 co‐positive cells using the manual method. In contrast, using the semi‐automated method, the time required was considerably reduced to approximately 4–5 h.

## 4 DISCUSSION

Simple assessment and subsequent prevention of skeletal muscle atrophy due to complications associated with aging and other chronic diseases are essential for maintaining individual health. However, diagnosing using histological observation requires lengthy analysis by a skilled pathologist. On the other hand, with the recent development of image analysis technology, attempts are being made to automate such pathological diagnosis. In particular, an automated platform has been developed to measure the CSA of skeletal muscle for each type of muscle fiber with an accuracy comparable to manual analysis (Babcock et al., 2020; Desgeorges et al., 2019; Mayeuf‐Louchart et al., 2018; Smith & Barton, 2014; Waisman et al., 2021). Despite the advancements in semi‐automating the analysis of numerous skeletal muscle fiber types, there has been limited exploration of semi‐automating the analysis of muscle differentiation marker expression (Lundquist et al., 2023; Rahmati & Rashno, 2021). In this study, we attempted to develop a semi‐automated method for quantifying Pax‐7‐positive cells to diagnose the regenerative capacity of skeletal muscle.

To validate the efficacy of the developed method, we orally administered A2, which has been demonstrated to induce muscle hypertrophy (Muta et al., 2023), to mice for 2 weeks and subsequently harvested the soleus muscles for utilization in the subsequent experiment.

Following repeated oral administration of A2, a slight increase in BrdU or BrdU/Pax‐7 positive cells was observed in mice's soleus muscles, whether the analysis was conducted manually or semi‐automatically, but not in Pax‐7 positive cells. A2 is known to be a polyphenol with potent physiological activity. A single oral administration of A2 increased the phosphorylation of AMP kinase and induced glucose transporter type 4 translocation in skeletal muscle (Yamashita et al., 2016). Furthermore, our previous study demonstrated that repeated oral administration of A2 suppressed proteolytic signals and promoted protein synthesis, resulting in reduced loss of soleus muscle weight in a mouse model of disuse atrophy (Muta et al., 2023). The present results showed that A2 increases the number of newly generated Pax‐7 positive cells and affects skeletal muscle differentiation. The mechanism of action of polyphenols, including A2, on skeletal muscle remains unknown, and further research is needed.

In all immunohistochemical analyses using the soleus muscles of mice administered A2, no difference was observed between the semi‐automated and manual methods (*p* > 0.999, Figure 3d–f). Many studies have previously established automated quantification methods for immunostained images of skeletal muscle fiber types, as mentioned above (Babcock et al., 2020; Desgeorges et al., 2019; Mayeuf‐Louchart et al., 2018; Smith & Barton, 2014; Waisman et al., 2021) as well as dystrophin (Sardone et al., 2018; Scaglioni et al., 2020; Vetter et al., 2022), a protein that causes muscular dystrophy. To employ these automated quantification methods clinically, the coefficient of determination *R* ^2^ between the manual and semi‐automated methods must be 0.8 or higher. In this experiment, an attempt was made to develop an automated quantification method for immunostained images of differentiation markers in skeletal muscle, and the *R* ^2^ was 0.7132 (Figure 4b). As shown in Figure 4a, it was suggested that the lower *R* ^2^ value might be due to variability in results between researchers using manual methods. To ascertain the practical utility of this method, it would be advantageous to re‐examine its validity using a disuse muscle atrophy model (Cornachione et al., 2014; Wan et al., 2016) or anaerobic exercise experiments (Farenia et al., 2019), in which the behavior of satellite cells undergoes a notable alteration. Although there is scope for enhancement in terms of precision, the semi‐automated quantitative approach was capable of measuring muscle differentiation markers in approximately one‐twentieth of the time required for manual procedures. This is a significant step forward in assessing the impact of multiple factors such as exercise and dietary factors.

In the present experiment, mice were pretreated with BrdU to evaluate the proliferation and differentiation potential of Pax‐7‐expressing satellite cells into muscle cells. However, a limitation of this study is that it is necessary to add skeletal muscle differentiation markers such as MyoD and myogenin (Kang & Krauss, 2010; Wang & Rudnicki, 2011) to comprehensively evaluate the regenerative potential of skeletal muscle. These evaluation methods may be semi‐automated by applying the similar methodology established in the present study.

## 5 CONCLUSION

This study proposed a semi‐automated method for the immunohistological observation of Pax7/BrdU‐positive cells, which function as a marker of skeletal muscle differentiation. The semi‐automated method was shown to significantly reduce analysis time when compared to the manual method, which is known to be time‐consuming. A high correlation was observed when comparing the regenerative capacity of skeletal muscle using satellite cell markers between semi‐automated and manual methods. This indicates that the semi‐automated method can be used in various studies. Future research is expected to focus on increasing the number of skeletal muscle markers and utilize this experimental system, which has been validated in the present study, to conduct a comprehensive analysis of skeletal muscle differentiation.

## AUTHOR CONTRIBUTIONS

Naomi Osakabe and Vittorio Calabrese conceived and designed the research; Yamato Yoshida, Kenta Shimizu, and Kenshin Iwasa performed the research and acquired the data, Yasuyuki Fujii analyzed and interpreted the data. Yamato Yoshida and Kenta Shimizuwrote the draft of the paper. Ursula M. Jacob, Tilman Fritsch, and Ali S. Abdelhameed contributed to the writing of the manuscript. Naomi Osakabe and Vittorio Calabrese provided the final review of the paper. All authors read and approved the final manuscript.

## FUNDING INFORMATION

This work was supported by JSPS KAKENHI (Grant Number 23H02166, 2023).

## CONFLICT OF INTEREST STATEMENT

The authors declare that they have no conflict of interest.

## ETHICS STATEMENT

The protocol of this study was approved by the Animal Experimentation Committee of Shibaura Institute of Technology (Permit Number: AEA23008, 2023). All experiments are strictly conducted according to ARRIVE 2.0 guidelines (https://arriveguidelines.org/arrive‐guidelines).

## Supporting information


Data S1.

## Data Availability

All data are available within the article, Supplementary Information—Data S1.
