# Supplementary material for: A semi‐automated observation approach to quantify mouse skeletal muscle differentiation using immunohistochemistry
Source: Physiol Rep. 2025 Apr 14;13(7):e70330. doi: 10.14814/phy2.70330 (PMC11994891; doi:10.14814/phy2.70330)
Supplement: Supplementary file 1 — Data S1. [file PHY2-13-e70330-s001.docx]

**Supplmental code**

1. Image processing

The code for the image processing methods using Fiji (<https://imagej.net/software/fiji/>, accessed on August 3rd, 2024) described in 2.4.1 to 2.4.3. was provided below.

//Close window for image analysis

close("*");

//Start recording analysis time

startTime = getTime;

//Designation of various foldersshowMessage("Select the folder that contains the dapi images");

openDir_dapi = getDirectory("choose a Directory");

showMessage("Select the folder that contains the Pax7 images");

openDir_Pax7 = getDirectory("choose a Directory");

showMessage("Select the folder that contains the BrdU images");

openDir_BrdU = getDirectory("choose a Directory");

showMessage("Select a folder to save the analysis results");

saveDir = getDirectory("choose a Directory");

print("processing・・・");

setBatchMode(true);

//Creating a List

list_dapi = getFileList(openDir_dapi);

list_Pax7 = getFileList(openDir_Pax7);

list_BrdU = getFileList(openDir_BrdU);

//Create a folder to save the analysis results

File.makeDirectory(saveDir+"Analysis_results/");

saveDir_images = saveDir + "Analysis_results/";

//Create an image storage location for the analyzed part of the image

//dapi

File.makeDirectory(saveDir_images+"Analysis_image_of_dapi/");

saveDirdapi =saveDir_images +"Analysis_image_of_dapi/";

//BrdU

File.makeDirectory(saveDir_images+"Analysis_image_of_BrdU/");

saveDirBrdU =saveDir_images +"Analysis_image_of_BrdU/";

//Pax7

File.makeDirectory(saveDir_images+"Analysis_image_of_Pax7/");

saveDirPax7 =saveDir_images +"Analysis_image_of_Pax7/";

//dapi and Pax7 merge

File.makeDirectory(saveDir_images+"Analysis_image_of_dapiPax7/");

saveDir_dapiPax7 = saveDir_images + "Analysis_image_of_dapiPax7/";

//dapi and BrdU merge

File.makeDirectory(saveDir_images+"Analysis_image_of_dapiBrdU/");

saveDir_dapiBrdU = saveDir_images + "Analysis_image_of_dapiBrdU/";

//dapi, Pax7 and BrdU merge

File.makeDirectory(saveDir_images+"Analysis_image_of_dapiPax7BrdU/");

saveDir_dapiPax7BrdU = saveDir_images +"Analysis_image_of_dapiPax7BrdU/";

//To check the contents of the array "list

//Array.show(list_dapi);

//Array.show(list_BrdU);

//Array.show(list_Pax7);

//Dapi_staining

for (i=0; i<list_dapi.length; i++)

{

open(openDir_dapi+list_dapi[i]);

run("Subtract Background...", "rolling=10");

run("8-bit");

run("Sharpen");

setOption("BlackBackground", false);

run("Options...", "iterations=1 count=1 black");

run("Auto Threshold", "method=Mean white");

run("Convert to Mask");

run("Erode");

run("Dilate");

run("Analyze Particles...", "size=0-infinity pixel show=Masks summarize");

//Save the processed image

name = getTitle();

dotIndex = lastIndexOf(name,".");

title = substring(name,0,dotIndex);

newname = title + "analyzed_dapi.tiff";

saveAs("tiff", saveDirdapi + newname);

close("*");

}

print("next is Pax7 expression");

//Save analysis results

selectWindow("Summary");

filename = "Summary_of_dapi" + ".csv";

saveAs("Results", saveDir_images + filename);

close("Summary_of_dapi.csv");

//Empty SUMMARY for next analysis

close("Summary");

close("*");

//Pax-7_staining

for (i=0; i<list_Pax7.length; i++)

{

open(openDir_Pax7+list_Pax7[i]);

run("Find Edges");

run("8-bit");

run("Enhance Contrast", "saturated=0.35 normalize");

run("Subtract Background...", "rolling=5");

setOption("BlackBackground", false);

run("Options...", "iterations=1 count=1 black");

run("Auto Threshold", "method=RenyiEntropy white");

run("Convert to Mask");

run("Close-");

run("Outline");

run("Fill Holes");

run("Erode");

run("Open");

run("Analyze Particles...", "size=30-150 pixel show=Masks summarize");

//Save the processed image

name = getTitle();

dotIndex = lastIndexOf(name,".");

title = substring(name,0,dotIndex);

newname = title + "analyzed_Pax7.tiff";

saveAs("tiff", saveDirPax7 + newname);

close("*");

}

print("next is BrdU expression");

//Save analysis results

selectWindow("Summary");

filename = "Summary_of_Pax7" + ".csv";

saveAs("Results", saveDir_images + filename);

close("Summary_of_Pax7.csv");

//Empty SUMMARY for next analysis

close("Summary");

close("*");

//BrdU_staining

for (i=0; i<list_BrdU.length; i++)

{

open(openDir_BrdU+list_BrdU[i]);

run("8-bit");

run("Sharpen");

run("Subtract Background...", "rolling=2.5");

run("Convolve...", "text1=[-1 -1 -2 -2 -2 -1 -1\n-1 -2 -2 4 -2 -2 -1\n-2 -2 4 8 4 -2 -2\n-2 4 8 16 8 4 -2\n-2 -2 4 8 4 -2 -2\n-1 -2 -2 4 -2 -2 -1\n-1 -1 -2 -2 -2 -1 -1\n] normalize");

run("Convolve...", "text1=[-1 -1 -2 -2 -2 -1 -1\n-1 -2 -2 4 -2 -2 -1\n-2 -2 4 8 4 -2 -2\n-2 4 8 16 8 4 -2\n-2 -2 4 8 4 -2 -2\n-1 -2 -2 4 -2 -2 -1\n-1 -1 -2 -2 -2 -1 -1\n] normalize");

run("Convolve...", "text1=[-1 -1 -2 -2 -2 -1 -1\n-1 -2 -2 4 -2 -2 -1\n-2 -2 4 8 4 -2 -2\n-2 4 8 16 8 4 -2\n-2 -2 4 8 4 -2 -2\n-1 -2 -2 4 -2 -2 -1\n-1 -1 -2 -2 -2 -1 -1\n] normalize");

run("Enhance Contrast...", "saturated=0.01 normalize");

setOption("BlackBackground", false);

run("Options...", "iterations=1 count=1 black");

run("Auto Threshold", "method=Intermodes white");

run("Convert to Mask");

run("Analyze Particles...", "size=2-20 pixel show=Masks summarize");

//Save the processed image

name = getTitle();

dotIndex = lastIndexOf(name,".");

title = substring(name,0,dotIndex);

newname = title + "analyzed_BrdU.tiff";

saveAs("tiff", saveDirBrdU + newname);

close("*");

}

print("next is merge of dapiPax7 expression");

//Save analysis results

selectWindow("Summary");

filename = "Summary_of_BrdU" + ".csv";

saveAs("Results", saveDir_images + filename);

close("Summary_of_BrdU.csv");

//Empty SUMMARY for next analysis

close("Summary");

close("*");

**2.** Quantification

The code for the automated quantification described in 2.5. using Fiji (<https://imagej.net/software/fiji/>, accessed on August 3rd, 2024) was provided below.

//Quantification of Pax-7 positive cells

list_analyzed_dapi = getFileList(saveDirdapi);

list_analyzed_Pax7 = getFileList(saveDirPax7);

for (i=0; i<list_analyzed_dapi.length; i++)

{

//Open parsed dapi

open(saveDirdapi+list_analyzed_dapi[i]);

analyzed_dapi_name = getTitle();

//Open parsed Pax-7

open(saveDirPax7+list_analyzed_Pax7[i]);

analyzed_Pax7_name = getTitle();

imageCalculator("AND create", analyzed_Pax7_name, analyzed_dapi_name);

run("Convert to Mask");

run("Dilate");

run("Analyze Particles...", "size=0-Infinity pixel show=Masks summarize");

run("Convert to Mask");

run("Dilate");

run("Dilate");

//Save the processed image

dapiPax7_name = getTitle();

dapiPax7_dotIndex = lastIndexOf(dapiPax7_name,".");

dapiPax7_title = substring(dapiPax7_name,0,dapiPax7_dotIndex);

dapiPax7_newname = dapiPax7_title + ".tiff";

saveAs("tiff", saveDir_dapiPax7 + dapiPax7_newname);

close("*");

}

//Save analysis results

selectWindow("Summary");

dapiPax7_filename = "Merge_summary_of_dapi_Pax7" + ".csv";

saveAs("Results", saveDir_images + dapiPax7_filename);

close("Merge_summary_of_dapi_Pax7.csv");

print("next is merge of dapiBrdU expression");

//Empty SUMMARY for next analysis

close("Summary");

close("*");

//Quantification of BrdU positive cells

list_analyzed_dapi = getFileList(saveDirdapi);

list_analyzed_BrdU = getFileList(saveDirBrdU);

for (i=0; i<list_analyzed_dapi.length; i++)

{

//Open parsed dapi

open(saveDirdapi+list_analyzed_dapi[i]);

analyzed_dapi_name = getTitle();

//Open parsed BrdU

open(saveDirBrdU+list_analyzed_BrdU[i]);

analyzed_BrdU_name = getTitle();

imageCalculator("AND create", analyzed_BrdU_name, analyzed_dapi_name);

run("Convert to Mask");

run("Median...", "radius=1");

run("Dilate");

run("Dilate");

run("Analyze Particles...", "size=0-Infinity pixel show=Masks summarize");

run("Convert to Mask");

run("Dilate");

run("Dilate");

run("Watershed");

//Save the processed image

dapiBrdU_name = getTitle();

dapiBrdU_dotIndex = lastIndexOf(dapiBrdU_name,".");

dapiBrdU_title = substring(dapiBrdU_name,0,dapiBrdU_dotIndex);

dapiBrdU_newname = dapiBrdU_title + ".tiff";

saveAs("tiff", saveDir_dapiBrdU + dapiBrdU_newname);

close("*");

}

//Save analysis results

selectWindow("Summary");

dapiBrdU_filename = "Merge_summary_of_dapi_BrdU" + ".csv";

saveAs("Results", saveDir_images + dapiBrdU_filename);

close("Merge_summary_of_dapi_BrdU.csv");

print("next is merge of dapiPax7BrdU expression");

//Empty SUMMARY for next analysis

close("Summary");

close("*");

//Quantification of Pax-7 and BrdU positive cells

list_analyzed_dapiPax7 = getFileList(saveDir_dapiPax7);

list_analyzed_dapiBrdU = getFileList(saveDir_dapiBrdU);

for (i=0; i<list_analyzed_dapiPax7.length; i++)

{

//Open parsed Pax-7 positive

open(saveDir_dapiPax7+list_analyzed_dapiPax7[i]);

analyzed_dapiPax7_name = getTitle();

//Open parsed BrdU positive

open(saveDir_dapiBrdU+list_analyzed_dapiBrdU[i]);

analyzed_dapiBrdU_name = getTitle();

imageCalculator("AND create", analyzed_dapiPax7_name,analyzed_dapiBrdU_name);

run("Analyze Particles...", "size=0-Infinity pixel show=Masks summarize");

//Save the processed image

dapiPax7BrdU_name = getTitle();

dapiPax7BrdU_dotIndex = lastIndexOf(dapiPax7BrdU_name,".");

dapiPax7BrdU_title = substring(dapiPax7BrdU_name,0,dapiPax7BrdU_dotIndex);

dapiPax7BrdU_newname = dapiPax7BrdU_title + ".tiff";

saveAs("tiff", saveDir_dapiPax7BrdU + dapiPax7BrdU_newname);

close("*");

}

//Save analysis results

selectWindow("Summary");

dapiPax7BrdU_filename = "Merge_summary_of_dapi_Pax7_BrdU" + ".csv";

saveAs("Results", saveDir_images + dapiPax7BrdU_filename);

close("Merge_summary_of_dapi_Pax7_BrdU.csv");

//Close All

close("Summary");

close("*");

setBatchMode(false); //Put the image being processed in a state to be displayed again

//End of analysis time recording

endTime = getTime;

elapsedTime = endTime - startTime;

elapsedTimeSeconds = elapsedTime / 1000;

print("Time taken for analysis: " + elapsedTimeSeconds + "Seconds");

//End of analysis

beep(); //Beep

print("analysis is completed");

showMessage("End of analysis");
